# Editorial: Beyond Borders: Myotonic Dystrophies–A European Perception

**DOI:** 10.3389/fneur.2018.00787

**Published:** 2018-09-20

**Authors:** Benedikt Schoser, Giovanni Meola

**Affiliations:** ^1^Department of Neurology, Friedrich-Baur-Institute, Ludwig-Maximilians-University Munich, Munich, Germany; ^2^Department of Biomedical Sciences for Health University of Milan, Milan, Italy; ^3^Department of Neurology, IRCCS Policlinico San Donato, Milan, Italy

**Keywords:** myotonic dystrophy type 1 and type 2, DM1, DM2, CNS involvement, repeat disorders, multisystemic diseases, cell models, animal models

Myotonic dystrophies (DMs) are pleotropic multisystemic diseases. These dominantly transmitted repeat disorders affect multiple organs of the human body at all ages—from the newborns to the elderly. DMs are highly inconsistent in terms of age at onset, severity of symptoms, and clinical patterns. Even within families, the onset and pattern of organ involvement remains enigmatic. Anticipation, with aggravation of the disease severity and earlier age at onset through successive generations, is particularly evident in DM1 that can affect adults and children at birth or during childhood 26 years ago, the identification of the DM1 repeat mutation in the DMPK gene on chromosome 19 opened the box for these diseases. The highly unstable CTG repeat expansion involved in DM1 usually increases from one generation to the next and is, to some extent, linked to disease severity. Clinically, patients with DM1 can be subdivided into five main classes, distinguishable by the prevalence of the presenting clinical pattern: congenital, childhood-onset, juvenile, adult-onset, and late-onset/asymptomatic. In myotonic dystrophy type 1 (DM1), the disease leads to a premature death, whereas in myotonic dystrophy type 2 (DM2) premature aging can be observed. In the past decades, much progress has been made in the pathomolecular understanding of the underlying DNA and RNA mechanisms of clinical miscellaneous DM symptoms. Presently, we are on the verge of transferring multiple bench-made molecular experimental therapies and knowledge into clinical therapeutic tools and reality, with the ultimate aim of alleviating and, eventually, curing the diseases. The present Research Topic represents a timely addition to the expanding body of evidence which aims to provide novel perspectives in our understanding of myotonic dystrophies. This collection of original contributions and standpoint reviews from multiple leading DM centers in Europe describes the state of the art for the characterization of the DMs disease, the development of molecular strategies to target its multisystemic nature, and provides evidence of screening and testing novel therapeutic avenues.

As an introduction to the current phenotype concept in myotonic dystrophy types 1 (DM1) and type 2 (DM2), Wenninger et al. summarize clinical core features of these highly variable subtypes.

Callus et al. focused on the neuropsychological and psychological assessment in a study on 31 DM1 patients. In 19.4% of DM1 patients a moderate or high level of symptoms intensity index is found. Fatigue and daytime sleepiness are associated with higher levels of psychoticism. Longer disease duration is associated with cognitive impairment evaluated through ENB-2 (*p* < 0.05). The need of neuropsychological and psychological screening and support for these patients and their families is addressed and a clinical protocol is advanced.

Minnerop et al. summarize the clinically important neuroimaging data. Structural gray and white matter abnormalities are seen in both DMs. In type 1, a consistent widespread cortical and subcortical involvement of gray and white matter affecting the whole brain is observed. Spectroscopy studies show neuronal and glial damage in both types. Correlative analyses of neuroimaging and clinical parameters stay diverse and are not well-reproducible. Latest insights argue more for disturbed networks as functional and structural substrates of the clinical symptoms. Longitudinal studies are required for future therapeutic studies.

Mahyera et al. report an estimated DM2 prevalence of 9 in 100,000, being as prevalent as DM1 in Germany. The expanded DM2 CCTG repeat tract comprises not only CCTG tetraplets but also repeated TG dinucleotides and TCTG tetraplet elements as well as NCTG interruptions. The normal allele sizes in the German population reveal that the CCTG repeat tract is usually interrupted by at least three tetraplets which is supposed to render stability. The largest analyzed normal allele has 23 uninterrupted CCTGs and may represent an instable early premutation allele. Their diagnostic results support a premutation range between 25 and 75 CCTGs, however clinical relevance of these premutation alleles are still uncertain. A fluid transition of penetrance is more likely as a clear cut-off of CCTG numbers in DM2.

Meinke et al. reflect on accelerated aging in DM. Clinical DM features are similar to aging aspects. Therefore DM could be classified as a segmental progeroid disease. However, molecular parallelism of accelerated aging in DM and segmental progeroid disorders are not reported yet. Now on cellular level molecular similarities to some progeroid syndromes of the nuclear envelope are detected. This first clinico-cellular comparison claims for the qualification of DM as a true segmental progeroid disorder.

Braz et al. summarize the modeling of myotonic dystrophy in mice, in order to provide investigational tools of the molecular and cellular pathogenesis. Mouse models are contributing intensely to our disease understanding. However, we still do not know how the molecular abnormalities described translate into CNS dysfunction. The authors review mouse models for neuromuscular aspects of disease, therapy development, and describe current limitations.

Chakraborty et al. discuss the molecular basis of cardiac dysfunction in myotonic dystrophies. Drosophila combines the acquiescence of its invertebrate genetics with the possibility of quickly acquiring physiological parameters. They review cardiac issues in both DMs, and the cardiac toxicity of non-coding CUG (DM1) and CCUG (DM2) repeat RNA in flies. Overexpression of muscleblind manages to strongly suppress arrhythmias and fractional shortening causing the cardiac phenotypes in flies. Small molecules pentamidine and daunorubicin are able to rescue cardiac phenotypes. Consequently, an assessment of candidate therapeutics in flies is possible.

André et al. review the pleiotropic problems of development, growth, regeneration, and aging of skeletal muscle. The molecular and cellular processes and roles of embryonic and adult muscle-resident stem cells in growth, homeostasis, regeneration, and premature aging is updated. Progenitor cells from extramuscular sources, such as pericytes and mesoangioblasts, participate in myogenic differentiation and may be of therapeutical potential for DM.

Matloka et al. review how cellular models decipher the molecular basis of DM1 and describe currently available cell models, ranging from exogenous expression of the CTG tracts to variable patients' derived cells.

Finally, López-Morató et al. report that therapeutic strategies for DM1 are mostly been focused on targeting CUGexpDMPK via reducing their expression and/or preventing interactions with MBNL1. Antisense oligonucleotides targeted to the CUG repeats in the DMPK transcripts are of particular interest due to their potential capacity to discriminate between mutant and normal transcripts. Nevertheless, alternative strategies using small molecule chemicals acting independently of a direct interaction with CUGexpDMPK are also reported. They summarize these chemicals and describe the beneficial effects in DM1 models. Moreover, they present potential mechanisms of action of these compounds and pathways they affect which could be considered for future therapeutic interventions in DM1.

As guest editors for this research topic on “Beyond borders: Myotonic dystrophies—a European perception,” we are delighted to commend to you the collection of 10 articles as an important contribution to the molecular and clinical medicine from Europe to the patients living with myotonic dystrophies.

## Author contributions

BS and GM conceived the manuscript. BS drafted the paper. GM critically appraised and edited the manuscript. Both authors read and approved the final version of the paper.

### Conflict of interest statement

The authors declare that the research was conducted in the absence of any commercial or financial relationships that could be construed as a potential conflict of interest.

